# Goreisan promotes diuresis by regulating the abundance of aquaporin 2 phosphorylated at serine 269 through calcium-sensing receptor activation

**DOI:** 10.1038/s41598-024-81324-y

**Published:** 2024-11-28

**Authors:** Keisuke Ogura, Naoki Fujitsuka, Miwa Nahata, Yohei Tokita

**Affiliations:** TSUMURA Kampo Research Laboratories, Research & Development Division, TSUMURA & CO., 3586 Yoshiwara, Ami-machi, Inashiki-gun, Ibaraki, 300-1192 Japan

**Keywords:** Goreisan, Aquaporin 2, Calcium-sensing receptor, CAMP, Kidney, Membrane trafficking

## Abstract

Aquaporin 2 (AQP2) contributes to water reabsorption and urine concentration by migrating to the luminal surface of the collecting ducts in an anti-diuretic hormone-stimulated manner, and the signaling pathway involved in AQP2 subcellular localization is a target for arginine vasopressin receptor antagonists (aquaretics). This study investigated the involvement of AQP2 in the diuretic effect and mechanisms of Goreisan (GRS), a traditional Japanese Kampo medicine used to treat conditions such as edema in patients with decreased urination. GRS exerted diuretic effects on desmopressin (DDAVP)-induced decreases in urine output and the level of AQP2 phosphorylated at Serine269 (pSer269-AQP2) in the renal tissues of mice. Furthermore, GRS inhibited the accumulation of pSer269-AQP2 to the luminal side following forskolin stimulation using a 3D culture model of the kidney collecting duct cell line mIMCD-3. GRS induced a transient increase in the intracellular Ca^2+^ concentration via the calcium-sensing receptor (CaSR) and suppressed the forskolin-stimulated increase in cAMP production. These results suggest that GRS regulates urine volume by modulating the subcellular localization of AQP2 via CaSR.

**Supplementary Information:**

The online version contains supplementary material available at 10.1038/s41598-024-81324-y.

## Introduction

Aquaporins (AQPs) are water-selective channels that contribute to water dynamics and homeostasis in the body. At present, 13 mammalian AQP isoforms have been identified^1,2^. Based on findings using deficient or mutant animals for each AQP isoform, dysfunction of AQP1, AQP2, and AQP3 expressed in the kidneys results in severely impaired urinary concentrating ability and polyuria^3–5^.

Goreisan (GRS, also known as “Wu-Ling-San” in China and “Oryeongsan” in Korea), a traditional Japanese Kampo medicine, is indicated for the relief of symptoms such as edema, nausea, diarrhea, and headache in patients with decreased urination. Decreased urine output has a variety of causes, and of these, heart failure induces edema with decreased urine because of fluid accumulation in the body caused by decreased cardiac function. Several clinical reports demonstrated that GRS increases urine output in patients with heart failure^6,7^, and a randomized clinical trial (GOREISAN-HF trial) with the primary endpoint of improving edema caused by heart failure is currently underway^8^. Several studies revealed that AQPs are involved in the effects of GRS. It is known that GRS can regulate urinary volume by modulating the gene expression of AQP1, AQP2, and AQP3 in partial nephrectomy and drug-induced kidney disease models^9,10^. In a lipopolysaccharide-induced diarrhea model, GRS ameliorated the inflammation-associated downregulation of AQP3 in the intestinal tract and improved diarrhea symptoms^11^. Furthermore, GRS exerts inhibitory effects on acute cerebral edema development caused by water intoxication by inhibiting the function of AQP4^12^.

In contrast to AQP3 and AQP4, which are continuously expressed on the basolateral membrane of renal collecting duct cells, the localization of AQP2 changes from the cytoplasm to the plasma membrane in response to stimulation with the anti-diuretic hormone vasopressin^13,14^. Therefore, to inhibit water reabsorption by AQP2, it is important to focus on the subcellular localization of AQP2 through arginine vasopressin receptor 2 (AVPR2)-dependent pathway activation.

Although it has been suggested that GRS regulates several AQPs, its effect on the subcellular localization changes of AQP2 in diuresis remains unclear. In this study, we investigated the diuretic effects of GRS on urine volume reduction via AVPR2 stimulation. In addition, we explored the mechanisms by which GRS regulates AQP2.

## Results

### Effect of GRS on urination

**Fig. 1:**
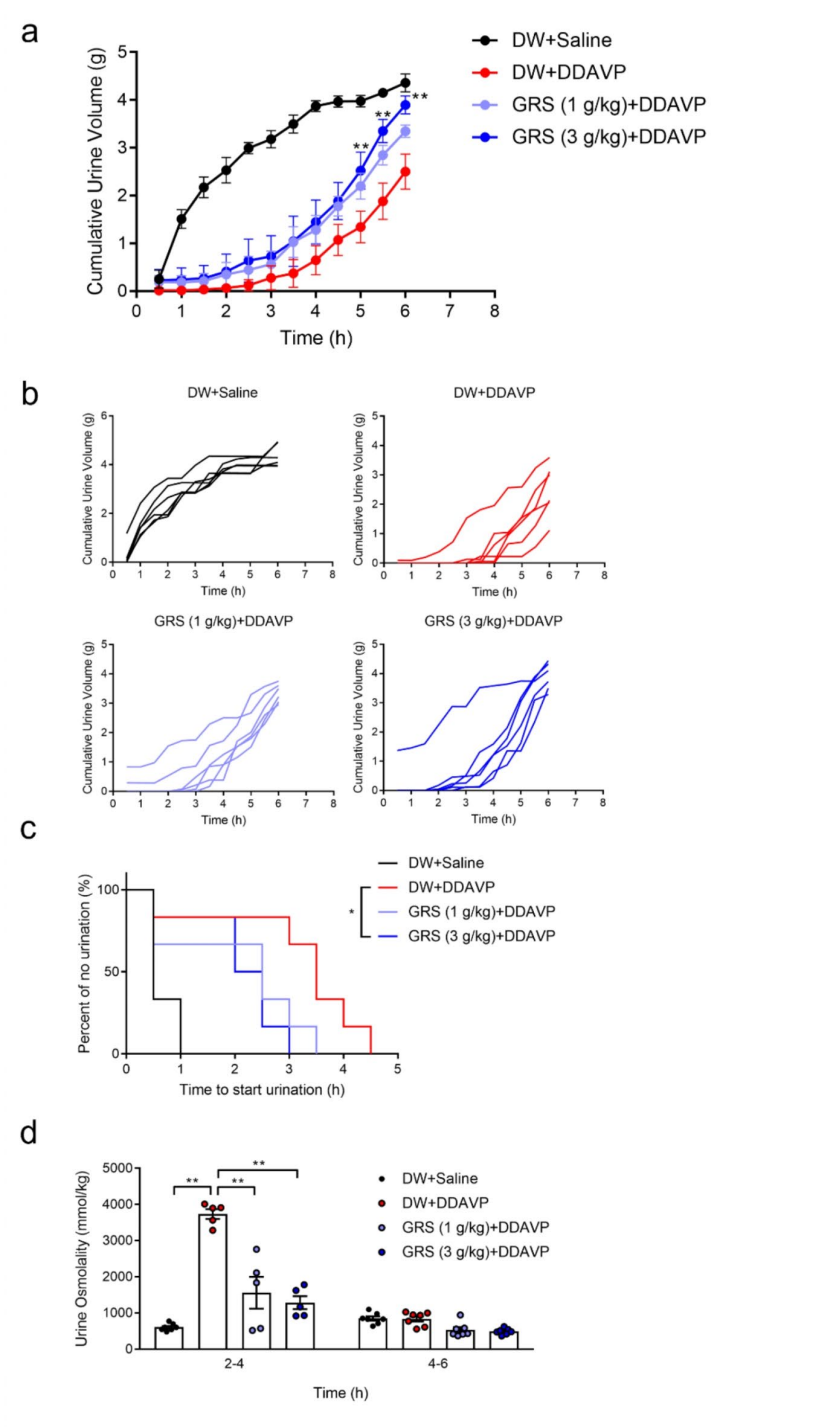
Effect of GRS on urinary output in DDAVP-treated mice. GRS (1–3 g/kg) or DW was orally administered to mice. Thirty minutes after oral administration, DDAVP (30 ng/kg) or saline was injected intraperitoneally at 90 mL/kg. Immediately after the intraperitoneal injection, mice were transferred to metabolic cages (three mice/cage, six cages), and urine output was measured every 30 min for up to 6 h. For urine osmolality measurements, mice were transferred to a metabolic cage (one mouse/cage), and urine was collected every 2 h for up to 6 h. (**a**) The average cumulative urine volume is presented as the mean ± SEM. (**b**) Cumulative urine volume of individual cages. (**c**) The time required to initiate urination is presented as the Kaplan–Meier curve. (**d**) Average urine osmolality is presented as the mean ± SEM and individual plots. Cumulative urine volume differences between groups were evaluated using two-way repeated-measures ANOVA, followed by Tukey’s multiple comparison test (**P < 0.01 vs. DW + DDAVP group). Differences in the urination start time between the DW + DDAVP and GRS (1 g/kg) + DDAVP groups or between the DW + DDAVP and GRS (3 g/kg) + DDAVP groups were evaluated using the log-rank test (*P < 0.025; Bonferroni’s correction). Differences in urine osmolality between the groups were evaluated using two-way repeated-measures ANOVA, followed by Tukey’s multiple comparison test (**P < 0.01).

The effect of GRS administration on the anti-diuretic effect of desmopressin (DDAVP) was investigated. The cumulative urine volume (three mice/cage) was measured in mice intraperitoneally treated with 90 mL/kg saline or an equivalent volume of DDAVP (30 ng/kg). Saline-treated mice urinated within 30 min after intraperitoneal administration, whereas DDAVP exerted an anti-diuretic effect immediately after administration. Meanwhile, 1 g/kg GRS tended to inhibit the anti-diuretic effect of DDAVP, whereas 3 g/kg GRS significantly suppressed the anti-diuretic effect of DDAVP and increased cumulative urine output at 5–6 h after DDAVP administration (*P* < 0.01; Fig. 1a), indicating a dose-dependent effect. At 6 h, approximately 40–60% of the amount of water loaded into the mice was excreted as urine. In addition, when the urine output of individual metabolic cages was evaluated, 3 g/kg GRS significantly shortened the time to the initiation of urination in DDAVP-treated mice (*P* < 0.025), whereas 1 g/kg GRS tended to shorten the time to urination without significance (Fig. 1b, c). Urine samples were collected from individual mice (*n* = 7) every 2 h to measure urine osmolality. Two hours after the start of urine collection, only a few DDAVP-treated mice were confirmed to have urinated (DW + DDAVP: *n* = 1, GRS (1 g/kg) + DDAVP: *n* = 2, GRS (3 g/kg) + DDAVP: *n* = 4). Compared with the effect of DDAVP alone between 2 and 4 h post-administration, GRS treatment significantly reduced urine osmolality (Fig. 1d). By 4–6 h after DDAVP administration, urine osmolality was comparable across all groups.

## Effect of GRS on the level of AQP2 phosphorylated at Ser269 (pSer269-AQP2)

**Fig. 2:**
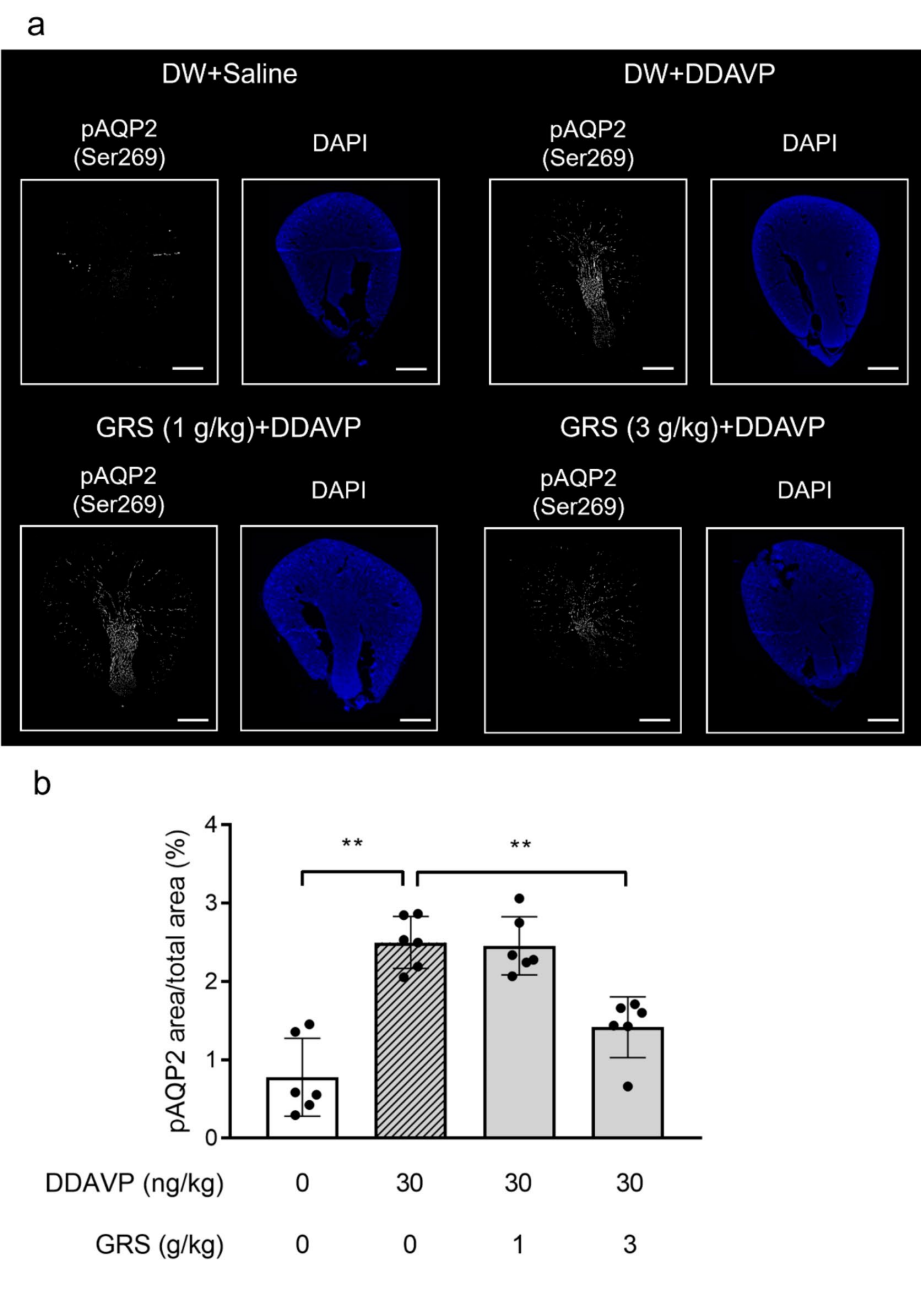
Effect of GRS on the phosphorylation of AQP2 at Ser269 in mouse kidneys. GRS (1–3 g/kg) or DW was orally administered to mice. Thirty minutes after oral administration, DDAVP (30 ng/kg) or saline was injected intraperitoneally at 90 mL/kg. Thirty minutes after the intraperitoneal injection, mice were perfusion-fixed with 4% paraformaldehyde, and kidneys were harvested. (**a**) Immunofluorescence staining images of phospho-AQP2 (pAQP2 [Ser269], white) and DAPI (blue) in renal tissue sections are presented. Scale bars, 1000 μm. (**b**) The ratio of the area of pAQP2 in kidney sections is presented as the mean ± SEM (*n* = 6). Differences between groups were evaluated using one-way ANOVA followed by a post-hoc Tukey’s multiple comparison test (**P < 0.01).

**Fig. 3:**
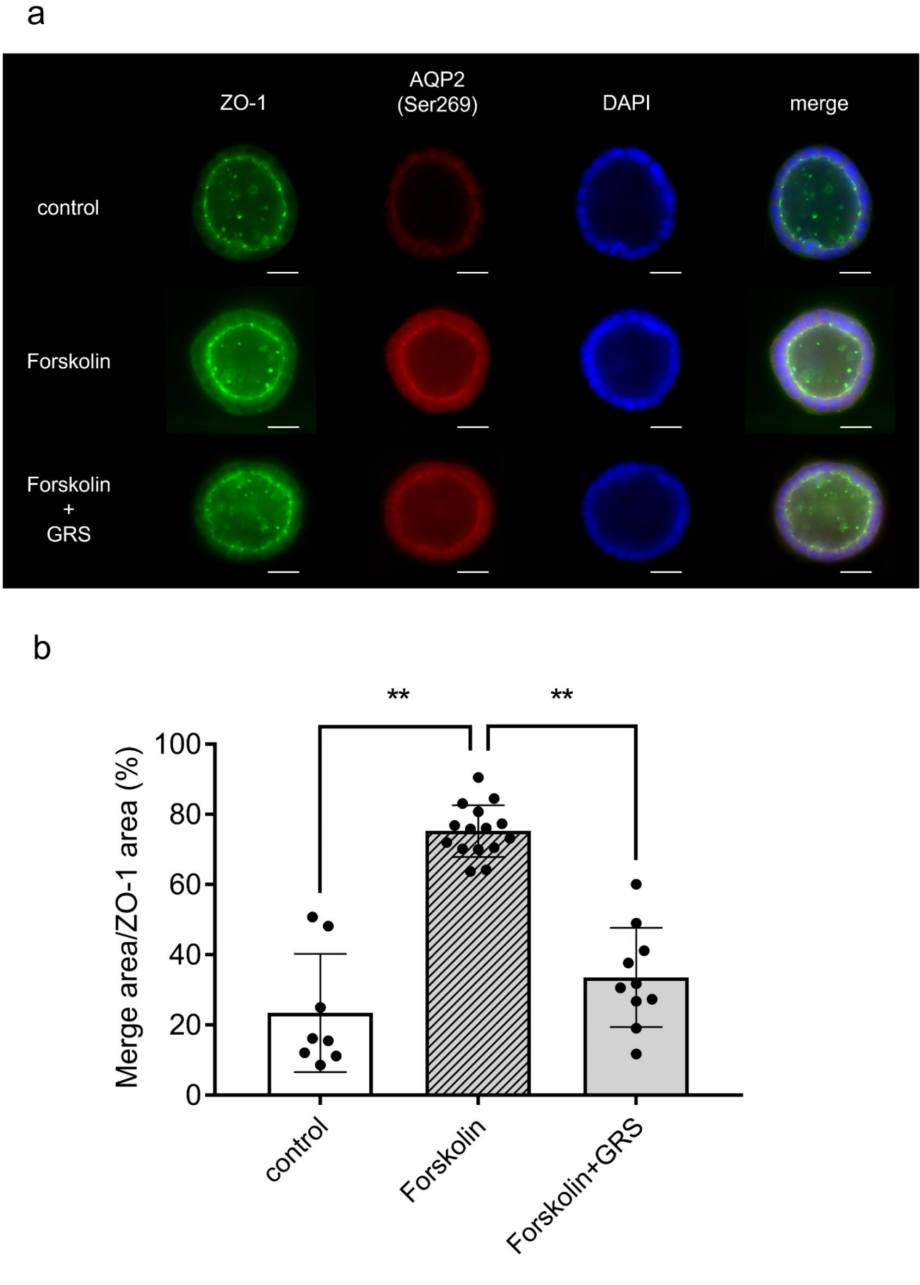
Effect of GRS on forskolin-induced AQP2 translocation in the mIMCD-3 spheroid culture model. mIMCD-3 cells were cultured in Matrigel for 7 days to permit spheroid formation. After medium replacement, GRS (100 µg/mL) was added to the culture medium. After 30 min of GRS treatment, spheroids were stimulated with forskolin (20 nM) for 1 h. Spheroids were fixed with 4% paraformaldehyde after forskolin stimulation. (**a**) Immunofluorescence staining of ZO-1 (green), phospho-AQP2 (pAQP2 [Ser269], red), and DAPI (blue) in spheroids is presented. Scale bars, 25 μm. (**b**) Colocalization area of ZO-1 and pAQP2 is presented as the mean ± SEM (control: *n* = 8, forskolin: *n* = 15, forskolin + GRS: *n* = 10). Differences between groups were evaluated using one-way ANOVA followed by a post-hoc Tukey’s multiple comparison test (**P < 0.01).

We investigated the effect of GRS on the phosphorylation status of AQP2, which is phosphorylated at Ser269 when AQP2 is stabilized at the apical membrane of the renal collecting ducts^15–17^. We confirmed the phosphorylation of AQP2 at Ser269 after DDAVP stimulation and its localization on the plasma membrane via co-staining for total AQP2, as previously reported^15–17^ (Supplementary Fig. 2). Conversely, pSer269-AQP2 was not detected in renal tissue from saline-treated mice, whereas pSer269-AQP2 levels were increased in mice at 30 min after DDAVP administration. At this time, 1 g/kg GRS had no significant effect on pSer269-AQP2 levels, whereas 3 g/kg GRS significantly reduced the pSer269-AQP2 levels in the kidney section area (*P* < 0.01; Fig. 2).To evaluate the amount of pSer269-AQP2 located on the luminal membrane, three-dimensional cultures of the mouse collecting duct cell line mIMCD-3 were used to form spheroids that recapitulated the cystic structure of renal collecting ducts. Consistent with previous findings^18,19^, the localization of the tight junction protein zonula occludens-1 (ZO-1) on the luminal membrane of the established spheroids was observed, confirming their luminal structure (Fig. 3a). Stimulation of spheroids with forskolin resulted in the accumulation of pSer269-AQP2 on the luminal membrane (ZO-1^+^ area). Meanwhile, 100 µg/mL GRS significantly reduced the co-localization of phosphorylated AQP2 and ZO-1 (Fig. 3a, b).

## Effect of GRS on forskolin-induced cAMP production in mIMCD-3 cells

**Fig. 4:**
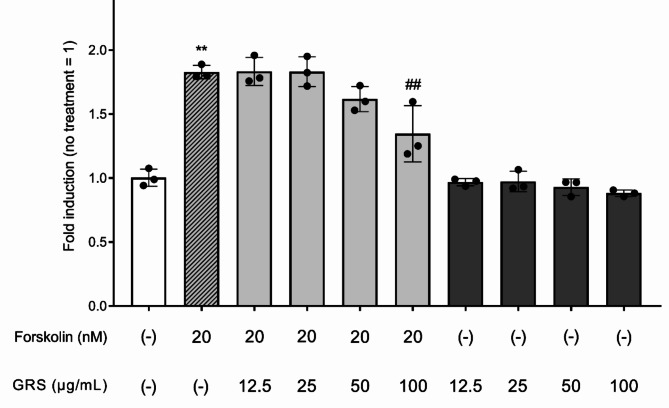
Effect of GRS on forskolin-induced cAMP production in mIMCD-3 cells. mIMCD-3 cells were seeded in a 96-well plate (2 × 10^4^ cells/well). Cells were treated with GRS (12.5–100 µg/mL) or vehicle for 15 min, followed by forskolin (20 nM) or vehicle for 15 min. Cells were lysed, and intracellular cAMP content was detected as the bioluminescence intensity. The luminescence of each group was normalized to that of the control (no treatment) group. The data in each column represent the mean ± SEM (*n* = 3). Differences between the control and forskolin treatment groups were evaluated using an unpaired *t*-test with Welch’s correction (**P < 0.01). Differences between the forskolin treatment group and each GRS treatment group were evaluated using one-way ANOVA followed by a post-hoc Dunnett’s multiple comparison test (##*P* < 0.01).

Forskolin stimulation increased cAMP production in mIMCD-3 cells (Fig. 4). Although GRS at concentrations up to 25 µg/mL did not inhibit this increase in cAMP production, a trend toward inhibition was observed at 50 µg/mL, and significant inhibition was recorded at 100 µg/mL (*P* < 0.01), demonstrating the concentration dependency of the effects of GRS on cAMP production. By contrast, the addition of GRS alone slightly but insignificantly inhibited cAMP production.

### Calcium-sensing receptor (CaSR)-mediated effects of GRS on intracellular Ca^2+^dynamics and forskolin-induced cAMP production in mIMCD-3 cells

**Fig. 5:**
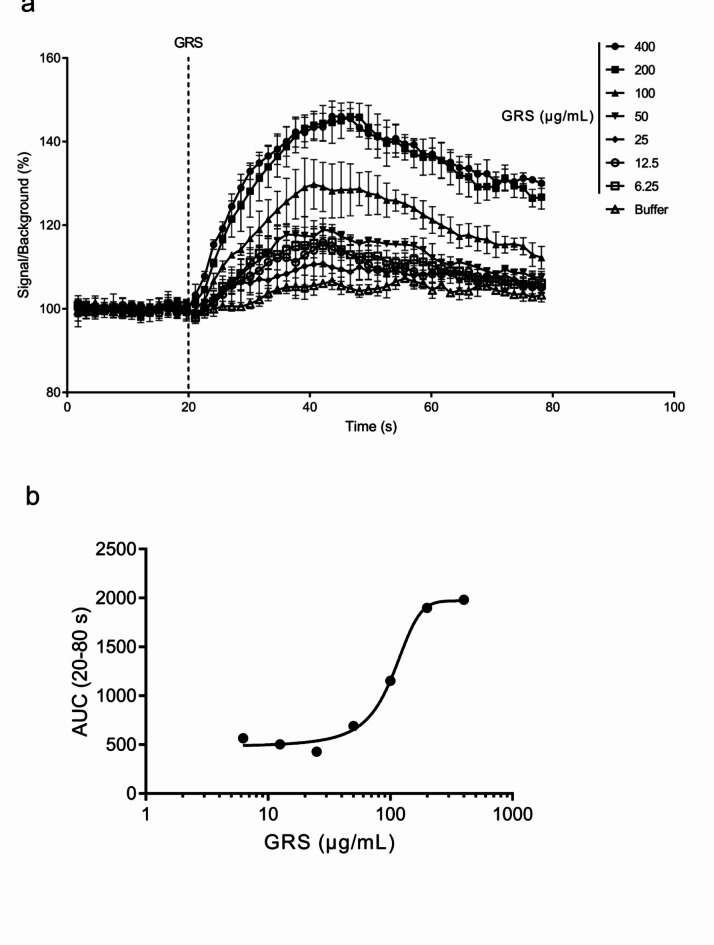
Effect of GRS on intracellular Ca^2+^dynamics in mIMCD-3 cells. mIMCD-3 cells (2 × 10^4^ cells/well in 96-well plate) were seeded and pretreated with Ca^2+^-chelating dye for 30 min. (**a**) Fluorescence intensity was measured for 80 s. Baseline fluorescence was measured for 20 s prior to the addition of GRS (6.25–400 µg/mL). (**b**) Changes in the intracellular Ca^2+^ concentration after the addition of GRS (20–80 s) were calculated as the AUC. Data in each column represent the mean ± SEM (*n* = 3).

A Ca^2+^ influx assay was performed to evaluate intracellular Ca^2+^ dynamics (Fig. 5a). The area under the curve (AUC) from GRS addition to the end of measurement (20–80 s) was used to quantify intracellular Ca^2+^ concentrations over time after GRS addition. The AUCs were increased by stimulation with GRS. A concentration–response relationship was observed (EC_50_ = 103.9 µg/mL), and a plateau was reached at 200 µg/mL GRS (Fig. 5b).

**Fig. 6:**
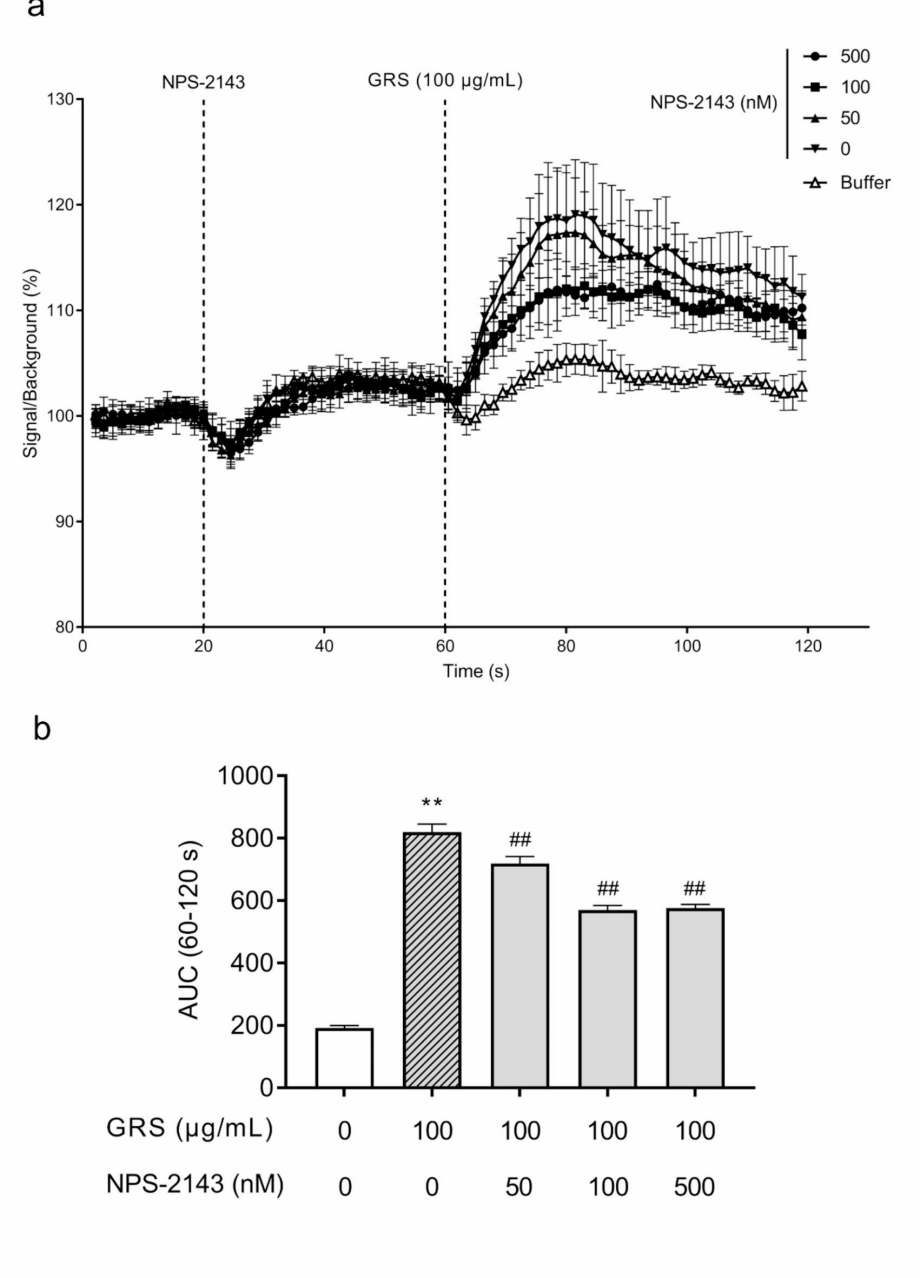
CaSR-mediated effect of GRS on intracellular Ca^2+^dynamics in mIMCD-3 cells. mIMCD-3 cells (2 × 10^4^ cells/well in 96-well plate) were seeded and pretreated with Ca^2+^-chelating dye for 30 min. (**a**) Fluorescence intensity was measured for 120 s. Baseline fluorescence was measured for 20 s prior to the addition of test compounds. NPS-2143 (50–500 nM) was added at 20 s, and GRS (100 µg/mL) was added at 60 s. (**b**) Changes in the intracellular Ca^2+^ concentration after the addition of GRS (60–120 s) were calculated as the AUC. Data in each column represent the mean ± SEM (*n* = 3). Differences between the control (no treatment) and GRS treatment groups were evaluated using an unpaired *t*-test with Welch’s correction (**P < 0.01). Differences between the GRS treatment group and each NPS-2143 treatment group were evaluated using one-way ANOVA followed by a post-hoc Dunnett’s multiple comparison test (##*P* < 0.01).

**Fig. 7:**
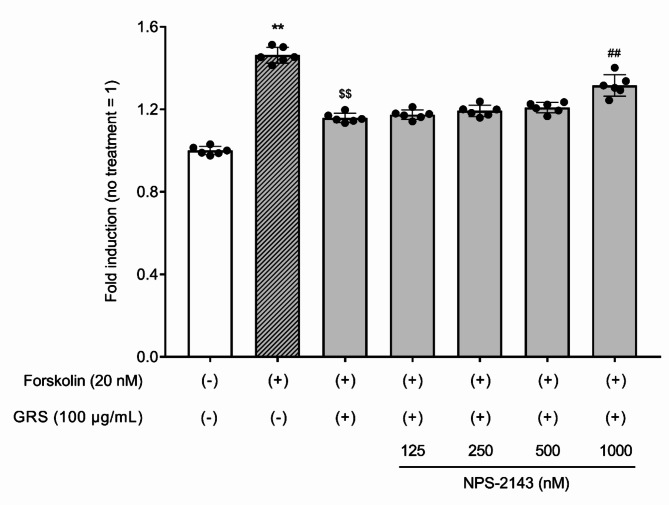
CaSR-mediated effect of GRS on forskolin-induced cAMP production in mIMCD-3 cells. mIMCD-3 cells were seeded in a 96-well plate (2 × 10^4^ cells/well). Cells were treated with GRS (100 µg/mL) ± NPS-2143 (125–1000 nM) or vehicle for 15 min, followed by forskolin (20 nM) or vehicle for 15 min. Cells were lysed, and the intracellular cAMP content was detected as the bioluminescence intensity. The luminescence of each group was normalized to that in the control (no treatment) group. Data in each column represent the mean ± SEM (*n* = 6). Differences between the control and forskolin treatment groups were evaluated using an unpaired t-test with Welch’s correction (**P < 0.01). Differences between the forskolin treatment group and each GRS ± NPS-2143 treatment group were evaluated using one-way ANOVA followed by a post-hoc Tukey’s multiple comparison test ($$*P* < 0.01: forskolin group vs. GRS group, ^##^*P* < 0.01: GRS group vs. GRS + NPS-2143 [1000 nM] group).

To investigate whether GRS-induced changes in intracellular Ca^2+^ levels are mediated by CaSR, cells were pretreated with the selective CaSR inhibitor NPS-2143. NPS-2143 pretreatment concentration-dependently inhibited the increase in intracellular Ca^2+^ levels induced by 100 µg/mL GRS (Fig. 6). The inhibitory effect of GRS (100 µg/mL) on cAMP production induced by 20 nM forskolin stimulation was partially antagonized in a concentration-dependent manner by pretreatment with NPS-2143 (Fig. 7).

## Discussion

In the present study, we found that GRS increased urine volume and decreased the abundance of pSer269-AQP2 in the kidneys of mice with decreased urine volume due to DDAVP administration. In addition, spheroid studies revealed that GRS inhibited the forskolin-induced accumulation of pSer269-AQP2 on the luminal membrane (ZO-1^+^ area). The mechanism of GRS was found to partially involve CaSR-mediated inhibition of increases in intracellular cAMP production. These results suggest that the diuretic effect of GRS is attributable to its ability to modulate the DDAVP-regulating subcellular localization of AQP2 via CaSR activation. To our knowledge, this is the first study to evaluate the effect of GRS on urinary volume regulation through the control of AQP2.

AQP2 is a water-selective channel expressed in renal collecting ducts. In response to stimulation via the G protein-coupled receptor vasopressin receptor 2, cytoplasmic AQP2 is phosphorylated at specific serine residues, and it exerts anti-diuretic effects by localizing to the luminal side of the collecting ducts and promoting water reabsorption^14,20^. In humans, mutations in AQP2 are associated with nephrogenic diabetes insipidus, a form of polyuria resulting from impaired urine concentrating ability, suggesting the importance of AQP2 in urine volume regulation^21^. Ser256, the critical phosphorylation site for AQP2 translocation to the plasma membrane, is phosphorylated in the cytoplasm before V2 receptor stimulation, and Ser256-phosphorylated AQP2 does not localize only at the plasma membrane^17^. Conversely, Ser269 is phosphorylated after V2 receptor or forskolin stimulation, and its phosphorylation is correlated with AQP2 localization on the plasma membrane^15–17,19^. On the apical plasma membrane, phosphorylation of Ser269 also stabilizes AQP2 and facilitates its retention on the plasma membrane by reducing its endocytosis^17^. DDAVP-treated mice exhibited decreased urine output, increased urine osmolality and increased pSer269-AQP2 levels in renal tissue (Figs. 1 and 2, Supplementary Fig. 2). Because Ser269 phosphorylation is well correlated with the apical membrane localization of AQP2^15,16,22^, the decrease in urine volume in this model suggests that it reflects the inhibition of water reabsorption via the decrease in the expression of AQP2, which functions as a water channel on the apical membrane. In the present study, GRS ameliorated the reduction in urination and increased urine osmolality in DDAVP-treated mice and decreased pSer269-AQP2 levels, suggesting that GRS is a promising treatment for symptoms of reduced urine volume through its ability to target AQP2. In this study, GRS administration shortened the onset time of urination, and a significant increase in urine volume was observed 5 h after administration (Fig. 1). Urination is generally initiated by the voiding reflex as the volume of urine in the bladder increases^23,24^. Given the inhibitory effect of GRS on the DDAVP-induced increase in urine osmolality, the shortened time to urination might partially reflect the increase in urine storage in the bladder with inhibition of water reabsorption following GRS administration. The effective doses at which GRS altered pSer269-AQP2 levels and urine osmolality differed (Figs. 1d and 2). This difference might result from the different time points at which urine osmolality and pSer269-AQP2 levels were measured, potentially influencing the detectable effects of GRS at varying doses. Additionally, at the time of urine osmolality measurement, a larger proportion of animals in the 3 g/kg GRS-treated group had urinated 2 h after DDAVP administration. This might reflect the early phosphorylation in the DDAVP group without GRS, which then does not occur in the GRS group. The discrepancy between the time to urination or changes in urine osmolality and the changes of pSer269-AQP2 levels is considered a limitation of the experimental method. This study did not include a GRS alone group because we wanted to evaluate the effect of GRS on the symptoms of decreased urine output despite the presence of fluid retention. Kurita et al. reported the effect of GRS on urine output in saline-loaded rats, in which a low GRS dose of 100 mg/kg significantly increased 24-h urine output^25^, and it is possible that GRS alone has a similar effect on pSer269-AQP2 levels and increases urine output. Investigation of the effects of GRS in normal animals is a subject for future study.

Changes in the phosphorylation and subcellular localization of AQP2 can also be achieved by directly increasing the amount of cAMP, the production of which is enhanced downstream of vasopressin signaling, via stimulation with forskolin^26,27^. Consistent with previous reports^18,19^, spheroids of mIMCD-3 cells formed luminal structures with tight junctions expressing ZO-1 protein, and pSer269-AQP2 accumulated at the apical membrane (ZO-1^+^ area) upon forskolin stimulation. We found that GRS inhibited forskolin-stimulated AQP2 accumulation at the apical membrane using spheroids (Fig. 3). Furthermore, GRS inhibited the forskolin-stimulated increase in cAMP production (Fig. 4). These results are consistent with the in vitro effects of GRS on the enhancement of cAMP production and the subcellular localization changes of AQP2 upon hypertonic stress^28^. In addition to the direct AVP2R-antagonizing effects of alisol A, alisol B, and alisol B O-23 acetate, which are previously reported GRS-containing components^29^, the present study demonstrated that the effect of GRS on AQP2 after forskolin stimulation is partially attributable to the AVP2R-independent regulation of AQP2 associated with increased cAMP production, suggesting that GRS targets AQP2 via multiple pathways. These findings are supported by a recent clinical report by Kakeshita et al., who revealed that GRS treatment reduced urinary cAMP levels and urinary AQP2 excretion and increased urine volume in patients with congestive heart failure and preserved ejection fraction receiving tolvaptan (AVPR2 antagonist) therapy^6^.

CaSR is a G_i_ protein-coupled GPCR that recognizes divalent cations, including extracellular Ca^2+^ions, aromatic amino acids, and polycations, and it has been implicated in the regulation of AQP2 subcellular localization and urine volume^30–32^. Dihydrotachysterol-induced hypercalcemia models exhibited polyuria and decreased AQP2 expression^33^. In clinical findings, urinary AQP2 was correlated with the severity of enuresis, characterized by nocturnal polyuria and hypercalciuria^34^, and the regulation of calcium intake by a low-calcium diet resulted in decreased urination via the regulation of AQP2 expression/transport^35^. By analyzing the spatiotemporal changes in intracellular Ca^2+^ concentrations, we demonstrated that GRS transiently increased intracellular Ca^2+^ concentrations in mIMCD-3 cells and that pretreatment with NPS-2143, a selective CaSR inhibitor, attenuated these effects (Figs. 5 and 6). Furthermore, NPS-2143 partially reversed the inhibitory effect of GRS on forskolin-induced increases in cAMP production (Fig. 7). Several previous studies demonstrated that extracellular Ca^2+^inhibits forskolin-mediated AQP2 translocation to the apical membrane via CaSR^31,36^. In addition to the direct inhibition of adenylate cyclase activation by G_i_ protein, these CaSR-mediated inhibitory effects on forskolin-induced increases in cAMP production were mediated by the inhibition of adenylate cyclase and/or activation of phosphodiesterase attributable to elevated intracellular Ca^2+^concentrations^31^, consistent with the CaSR-mediated effects of GRS on elevated intracellular Ca^2+^ concentrations and suppression of cAMP production observed in this study. The affinity of CaSR for Ca^2+^ is 0.5–10 mM, which is in reasonable agreement with the physiological extracellular Ca^2+^concentration range^37,38^. The measured Ca^2+^ concentrations used in our in vitro assays were below the detection limit of 0.05 mM (data not shown), excluding the possibility that the CaSR-mediated response by GRS is attributable to calcium ions themselves. Although we were detected the influence of NPS-2143 on the effects of GRS in a highly sensitive assay system using intracellular Ca^2+^ concentrations and cAMP as indicators, we were technically unable to detect a significant influence of NPS-2143 on the effects of GRS on AQP2 subcellular localization in the spheroid-based study. This is an issue for future study.

The diuretic effect of GRS demonstrated in this study is attributable to the CaSR-mediated regulation of pSer269-AQP2 levels in response to various stimuli. Conversely, GRS has been demonstrated to exert anti-inflammatory and anti-fibrotic effects on renal injury with renal substrate changes attributable to 5/6 nephrectomy and drug-induced inflammation and fibrosis and to improve urine concentrating ability by correcting the changes in AQP2 gene expression^9,10^. It has been reported that CaSR is involved in both the subcellular localization of AQP2 and the regulation of gene and protein expression via microRNA and/or the ubiquitin–proteasome axis^39^. In the future, it will be important to investigate whether the AQP2-mediated effects of GRS are attributable to both membrane translocation and tissue repair effects or quantitative changes to develop treatment strategies based on the patient background.

Concerning the effect of plant-derived components on CaSR, olive leaf extract, which contains several bioactive components including polyphenols, has been reported to regulate the subcellular localization of AQP2 via CaSR^40^. Recently, ligand searches for herbal drug components based on binding affinity for CaSR and docking simulations revealed that several flavonoids, phenolic acids, and triterpenes act as ligands and inhibit calcium oxalate accumulation in the kidneys in an ethylene glycol-induced nephrolithiasis model^41–43^. GRS is a Kampo medicine consisting of five crude drugs including the medicinal parts of plants and sclerotia, but the ingredient-derived component responsible for the effects observed in this study is unclear. Further studies are needed, including identification of the active components of GRS and pharmacokinetic analyses.

In conclusion, we demonstrated that GRS exerts a diuretic effect on mice with DDAVP-induced reductions in urine output. This effect was partially attributable to the CaSR-mediated inhibition of increased cAMP production and modulation of DDAVP-regulated AQP2 subcellular localization. Our findings suggest that GRS exerts its diuretic effects through a mechanism that bypasses the AVPR2-dependent signaling pathway, and these findings are important for the development of new therapeutic approaches targeting body water regulation for patients with decreased urine output, including combination strategies with existing diuretics.

## Materials and methods

### Drugs and reagents

The powdered extract of GRS (Lot No. 2170017010) was supplied by Tsumura & Co. (Tokyo, Japan). GRS consists of five constituent crude drugs: 4 g of *Alisma* tuber (tuber of *Alisma orientale* Juzepczuk), 3 g of *Atractylodes lancea* rhizome (rhizome of *Atractylodes lancea* De Candolle), 3 g of *Polyporus* sclerotium (sclerotium of *Polyporus umbellatus* Fries), 3 g of *Poria* sclerotium (sclerotium of *Poria cocos* Wolf), and 1.5 g of cinnamon bark (*Cinnamomum cassia* Blume). The mixture of the five crude drugs was extracted with water at 95 °C for 1 h. The extraction solution was separated from the insoluble waste and concentrated by removing water under reduced pressure. The solution was spray-dried to obtain 2 g of dry extract powder. The detailed manufacturing process information is available on the website of Tsumura & Co. (https://www.tsumura.co.jp/english/kampo/manufacturing-process/).

GRS was approved by the Ministry of Health, Labour and Welfare of Japan, and its quality and safety were certified by Tsumura & Co. in accordance with the Pharmaceutical Affairs Act. DDAVP, forskolin, and NPS-2143 were purchased from Sigma-Aldrich (St. Louis, MO, USA).

### Animals

Male C57BL/6J mice were purchased from Jackson Laboratories Japan (Yokohama, Japan) at 6 weeks of age and used from 7 weeks of age after habituation. All mice were maintained at a temperature of 23 ± 3 °C and relative humidity of 50% ± 20% under a 12-h/12-h light–dark cycle. Mice were permitted free access to food and water.

### Measurement of urine output

The timeline of the in vivo experimental design is presented as Supplementary Fig. 1. Seventy-two mice were randomly divided into four groups: distilled water (DW) + saline, DW + DDAVP, GRS (1 g/kg) + DDAVP, and GRS (3 g/kg) + DDAVP (18 mice/group). After overnight starvation, the mice were orally administered DW or GRS suspended in DW at a concentration of 10 mL/kg. The doses of GRS (1 and 3 g/kg) were estimated according to previous animal studies^11,12,44^. To increase urinary output in mice, 90 mL/kg saline was injected intraperitoneally 30 min after oral administration. DDAVP (30 ng/kg) was dissolved in saline. Immediately after drug administration, mice were transferred to metabolic cages (three mice/cage) with free access to water, and the cumulative urine output of individual cages was measured every 30 min for up to 6 h. At the end of the experiment, mice were euthanized via cervical dislocation.

### Measurement of urine osmolality

Twenty-eight mice were randomly divided into four groups (seven mice/group) and treated as described in the Measurement of urine output section. Immediately after drug administration, mice were transferred to metabolic cages (one mouse/cage) with free access to water, and urine samples were collected every 2 h for up to 6 h to measure urine osmolality using a vapor pressure osmometer (Xylem ELITech 5600, Xylem Inc. Washington, DC, USA). At the end of the experiment, mice were euthanized via cervical dislocation.

### Immunofluorescence staining of mouse kidneys

Under deep anesthesia with sevoflurane (3–4%), mice were euthanized via blood loss with saline perfusion through the left ventricle. Mice were then fixed by reperfusion with 4% paraformaldehyde (Muto Pure Chemicals Co., Ltd., Tokyo, Japan) after saline perfusion. Kidney tissue was collected and soaked in 4% paraformaldehyde overnight. Tissues were immersed in 10% sucrose solution for 24 h, followed by 20% sucrose solution for 24 h at 4 °C, and then embedded by OCT compound (Sakura Finetek, Tokyo, Japan). Frozen sections were prepared and reacted with rabbit anti-total AQP2 (dilution, 1:500, ab199975, Abcam, Cambridge, UK), goat anti-total AQP2 (dilution, 1:400, NBP1-70378, Novus Biologicals, Centennial, CO, USA), and/or rabbit anti-phospho-AQP2 (Ser269, dilution, 1:500, ab254072, Abcam) overnight at 4 °C, followed by Alexa Fluor 488-conjugated donkey anti-rabbit IgG (dilution, 1:1000, A21206, Invitrogen, Thermo Fisher Scientific, Waltham, MA, USA), Alexa Fluor 546-conjugated goat anti-rabbit IgG (dilution, 1:1000, A11035, Invitrogen) and/or Alexa Fluor 546-conjugated donkey anti-goat IgG (dilution, 1:1000, A10040, Invitrogen) at room temperature for 2 h. After nuclear staining with DAPI (Invitrogen), the tissue was mounted with Fluoromount/Plus (Diagnostic BioSystems, Pleasanton, CA, USA). Immunofluorescence images were obtained using a BZ-X710 fluorescence microscope (Keyence, Osaka, Japan). The total area of kidney tissue sections and the phospho-AQP2–positive areas were calculated using ImageJ software (US National Institutes of Health, Bethesda, MD, USA).

### Cell culture

mIMCD-3 cells were purchased from ATCC (Manassas, VA, USA, cat. no. CRL-2123). Cells were cultured in DMEM: F-12 medium (contains 2.5 mM l-glutamine, 15 mM HEPES, 0.5 mM sodium pyruvate, and 1200 mg/L sodium bicarbonate, ATCC) supplemented with 100× penicillin–streptomycin solution (FUJIFILM Wako Pure Chemical, Tokyo, Japan) and 10% fetal bovine serum (Gibco, Thermo Fisher Scientific) under 5% CO_2_ and 37℃.

### Culture of spheroids

Spheroid culture was performed according to the protocol of Rachel et al.^18^. Briefly, mIMCD-3 cell suspensions prepared at a density of 1 × 10^5^ cells/mL in DMEM: F-12 were mixed with an equal volume of growth factor-reduced Matrigel (BD Biosciences, Franklin Lakes, NJ, USA) and seeded into eight-well coverglass chamber slides (Iwaki, Tokyo, Japan) at a volume of 200 µL per well. The gel was incubated at 37 °C and 5% CO_2_ until it solidified, and 200 µL of medium were added. Cells were cultured for 7–10 days with medium changes every 24 h. Spheroid formation with a luminal structure was visually confirmed, and the spheroids were used for subsequent analysis. The same threshold was set for all acquired images during the binarization process. ZO-1^+^ and phospho-AQP2^+^ areas were calculated for the spheroids, and the merged area was selected and calculated. Image analysis was performed using ImageJ software.

### Immunofluorescence staining of spheroids

Fluorescent immunostaining of spheroids was performed as described by Rachel et al.^18^. Briefly, spheroid culture gels were washed with DPBS (Gibco), 4% paraformaldehyde phosphate buffer solution (FUJIFILM Wako Pure Chemical) was added, and the gels were incubated on a shaker at room temperature for 30 min. After the spheroids were sedimented, they were washed with DPBS, and permeabilization buffer (prepared by adding 7 mg/mL gelatin from porcine skin [Sigma-Aldrich] and 0.5% Triton X-100 [Sigma-Aldrich] to DPBS) was added and allowed to react for 30 min at room temperature. Primary antibodies (rat anti-mouse ZO-1 [dilution, 1:500, sc-33725, Santa Cruz, Dallas, TX, USA], rabbit anti-phospho-AQP2 [Ser269, dilution, 1:500, ab254072, Abcam]) were reacted overnight at 4 °C, followed by secondary antibodies (Alexa Fluor 488-conjugated goat anti-rat IgG [dilution, 1:400, A11006, Invitrogen] an Alexa Fluor 546-conjugated goat anti-rabbit IgG [dilution, 1:400]) at room temperature for 4 h. After nuclear staining with DAPI and encapsulation with Fluoromount/Plus, the samples were observed under a BZ-X710 fluorescence microscope.

### Analysis of cAMP production

The increase in intracellular cAMP levels following forskolin stimulation was analyzed using the cAMP-Glo assay kit (Promega, Madison, WI, USA). mIMCD-3 cells (2 × 10^4^) were reacted with forskolin for 15 min and then incubated with cell lysis buffer for 15 min at room temperature on a shaker. In the case of GRS pretreatment, cells were incubated with GRS for 15 min prior to forskolin stimulation. The cell lysate was reacted with cAMP detection reagent, and luminescence was measured using a plate-reading luminometer (Infinite M Plex, TECAN, Männedorf, Switzerland).

### Ca^2+^ influx assay

The Ca^2+^influx assay was performed using the FLIPR calcium assay 5 kit (Molecular Devices, Sunnyvale, CA, USA) in accordance with a previously reported method^45^. Briefly, cells were seeded at 2 × 10^4^ cells/well in 96-well plates and incubated with 80 µL of Ca^2+^-chelating dye dissolved in assay buffer (1× HBSS, 20 mM HEPES, pH 7.4) for 30 min at room temperature. A FlexStation 3 microplate reader (Molecular Devices) was used to measure the fluorescence values of the plates over time. Fluorescence intensity was measured for a maximum of 120 s. Baseline fluorescence was measured for 20 s prior to the addition of test compounds. For CaSR inhibition studies, NPS-2143 was added at 20 s, and GRS was added at 60 s. Changes in the intracellular Ca^2+^ concentration after the addition of GRS were calculated as the AUC. The concentration–response curve was fitted to the sigmoidal dose–response equation, and EC_50_ was calculated. These calculation processes were performed using GraphPad Prism 7 software (Boston, MA, USA).

### Statistical analysis

Data are presented as Kaplan–Meier curves or the mean ± standard error of the mean (SEM). The statistical significance of differences was assessed using the log-rank test, unpaired *t*-test with Welch’s correction, one-way analysis of variance (ANOVA), or two-way repeated-measures ANOVA followed by a post-hoc Dunnett’s multiple comparison test or Tukey’s multiple comparison test. Significance was indicated by *P* < 0.025 (log-rank test, Bonferroni’s correction) or *P* < 0.05 (unpaired *t*-test with Welch’s correction, Dunnett’s test, and Tukey’s test). Statistical analyses were conducted using GraphPad Prism 7 software.

## Electronic supplementary material

Below is the link to the electronic supplementary material.

Supplementary Material 1

## Data Availability

All data generated or analyzed during this study are included in this published article.
