# Supplementary material for: Goreisan promotes diuresis by regulating the abundance of aquaporin 2 phosphorylated at serine 269 through calcium-sensing receptor activation
Source: Sci Rep. 2024 Nov 28;14:29650. doi: 10.1038/s41598-024-81324-y (PMC11605074; doi:10.1038/s41598-024-81324-y)

**Supplementary Figure**

**Supplementary Figure 1. Timeline of the *in vivo* experimental design.**

The schedules of the cumulative urine volume and urine osmolality evaluations (urine volume: blue arrows, urine osmolality: orange arrows) and the kidney tissue sample collection for immunofluorescence staining (red arrows) are presented.


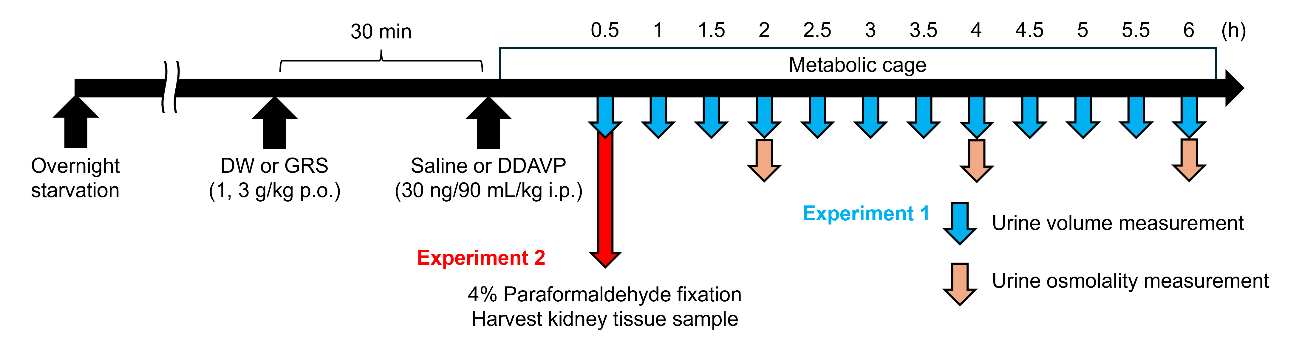


**Supplementary Figure 2. AQP2 phosphorylation and accumulation at the apical plasma membrane after DDAVP stimulation**

DDAVP (30 ng/kg) or saline was injected intraperitoneally. Thirty minutes after the intraperitoneal injection, mice were perfusion-fixed with 4% paraformaldehyde, and kidneys were harvested. (**a**) Immunofluorescence co-staining images of total AQP2 (red), phospho-AQP2 (pAQP2 [Ser269], green), and DAPI (blue) in renal tissue sections (DDAVP-treated group) are presented. (**b**) Immunofluorescence staining images of total AQP2 (red), pAQP2 (Ser269, red), and DAPI (blue) in renal tissue sections are presented. Scale bars, 10 μm.


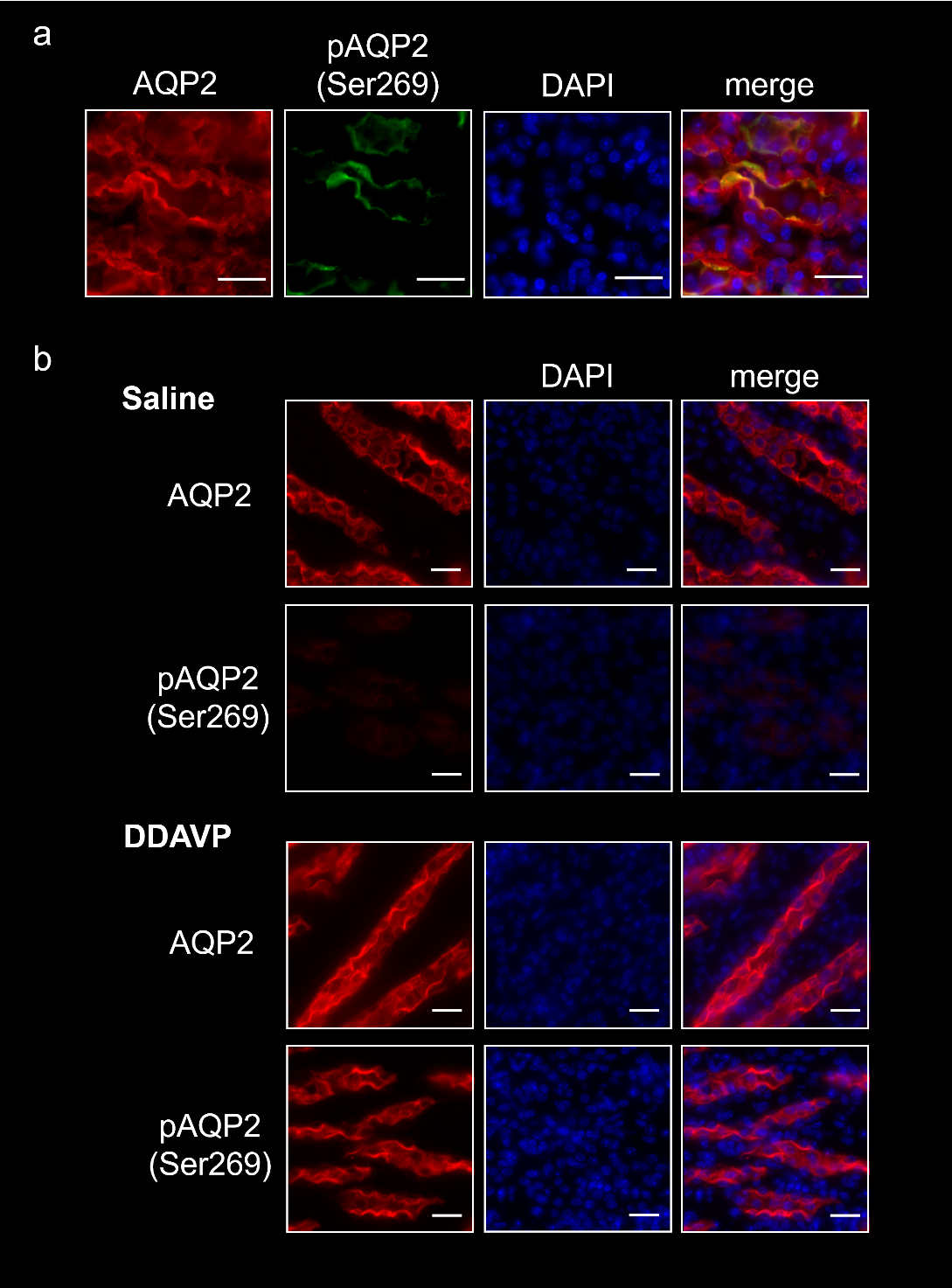

Supplement: Supplementary file 1 — Supplementary Material 1 [file 41598_2024_81324_MOESM1_ESM.docx]
